# Exploring the determinants of under-five mortality and morbidity from infectious diseases in Cambodia—a traditional and machine learning approach

**DOI:** 10.1038/s41598-024-70839-z

**Published:** 2024-08-27

**Authors:** Daniel Helldén, Serey Sok, Alma Nordenstam, Nicola Orsini, Helena Nordenstedt, Tobias Alfvén

**Affiliations:** 1 Department of Global Public Health, Karolinska Institutet, Tomtebodavägen 18 A, 171 77 Stockholm, Sweden; 2 Astrid Lindgren Children’s Hospital, Karolinska University Hospital, Stockholm, Sweden; 3 Research Office, Royal University of Phnom Penh, Phnom Penh, Cambodia; 4 Department of Medicine and Infectious Diseases, Danderyd University Hospital, Stockholm, Sweden; 5 Sachs’ Children and Youth Hospital, Stockholm, Sweden

**Keywords:** Infectious diseases, Risk factors, Paediatrics, Public health

## Abstract

Cambodia has made progress in reducing the under-five mortality rate and burden of infectious diseases among children over the last decades. However the determinants of child mortality and morbidity in Cambodia is not well understood, and no recent analysis has been conducted to investigate possible determinants. We applied a multivariable logistical regression model and a conditional random forest to explore possible determinants of under-five mortality and under-five child morbidity from infectious diseases using the most recent Demographic Health Survey in 2021–2022. Our findings show that the majority (58%) of under-five deaths occurred during the neonatal period. Contraceptive use of the mother led to lower odds of under-five mortality (0.51 [95% CI 0.32–0.80], p-value 0.003), while being born fourth or later was associated with increased odds (3.25 [95% CI 1.09–9.66], p-value 0.034). Improved household water source and higher household wealth quintile was associated with lower odds of infectious disease while living in the Great Lake or Coastal region led to increased odds respectively. The odds ratios were consistent with the results from the conditional random forest. The study showcases how closely related child mortality and morbidity due to infectious disease are to broader social development in Cambodia and the importance of accelerating progress in many sectors to end preventable child mortality and morbidity.

## Introduction

Cambodia has substantially improved child health over the last decades, with under-five mortality falling from 117 deaths per 1000 live births to 29 deaths per 1000 live births between 1990 and 2015^1^. Indeed, Cambodia was one of the few low- and middle-income countries that reached the Millennium Development Goal 4 by reducing the under-five mortality rate by two-thirds during this time^1^. In 2015 Cambodia adopted the ambitious 17 Sustainable Development Goals and contextualized them into the 18 Cambodian Sustainable Development Goals (with one additional goal for ending the negative impact of Mine/Explosive remnants of war)^2^. The country has made significant strides towards a range of the SDGs^3^, and in 2021 the under-five mortality rate was estimated to be 25 per 1000 live births effectively reaching the SDG 3.2 target on child mortality^4^. Child mortality, and in particular the neonatal mortality, remain high in Cambodia comparison with other countries in Southeast Asia^4^. The advancement in child health in Cambodia has been attributed to multisectoral efforts, and cooperation between different sectors has been key in implementing child health strategies^2,5,6^. A few studies have tried to decipher the most important variables for under-five mortality in Cambodia, utilizing parametric statistical analysis and with data from before 2015 and with relatively different results. Using data from the Demographic Health Surveys (DHS) conducted in 2010 and 2014, Ly et al.^7^ concluded that longer childbirth intervals, maternal antenatal care visit as last birth, and children being fully vaccinated were associated with lower risk of mortality, while older maternal age and higher education level of the mother were associated with higher risk of mortality. Focusing on the data from the 2014 DHS, Um and Heng^8^ used logistic regression to showcase that children with lower birth weight and living in rural areas had a higher risk of mortality while those born to mothers who use contraceptives had a lower risk of mortality.

Neonatal disorders and infectious diseases continue to cause the most disability-adjusted life years for children under five years in Cambodia according to the global burden of disease^9^. For children aged 5–14 years, the causes of morbidity are more varied but are primarily caused by non-communicable diseases and injuries^9^. The mortality rate among under-five children from lower respiratory infections in Cambodia has declined by more than 80% since 1990, mainly due to increased vaccination coverage, lower household air pollution, and better nutritional status of children^10^. Nonetheless, lower respiratory infections are still the leading infectious cause of death and morbidity, with diarrhea as the second^9^. The possible drivers of lower respiratory infections in children in Cambodia have not been studied, however a study by Vong et al.^11^ using parametric statistical analysis of 2014 DHS data showed that lack of water and sanitation facilities and maternal unemployment was associated with higher risk of diarrhea while older maternal age was associated with a lower risk of diarrhea.

As described in the above studies on child mortality and morbidity in Cambodia^7,8,11^, as well as other global estimates of determinants of child health^12,13^, standard linear, logistical or a mix thereof has been the only statistical approach used. These methods might be limited when the data to be analyzed has a high degree of correlation, random noise or does not follow assumptions of normality. However, machine learning has been shown to be provide complementary evidence on the determinants of child health in low- and middle-income countries. For instance, Bizzago et al.^14^ used a random forest with data from household surveys in 27 countries to assess the most important determinants of under-five mortality, while Methund et al.^15^ showed how a logistic classifier machine learning algorithm could be used to explore determinants of infectious diseases in children from a multiple indicator cluster survey.

Overall, Cambodia has shown a continued reduction in the under-five mortality rate and child morbidity from infectious diseases since 2015. However, no study has investigated child mortality and morbidity data after 2015 or applied machine learning which has emerged as a useful approach to complement more traditional parametric statistical analyses. Hence, the aim of this study was to explore factors that might be associated with under-five mortality and child morbidity from infectious diseases using the most recent DHS conducted in Cambodia.

## Methods

### Data source

This study is based on quantitative data derived from the Cambodian DHS (CDHS), a nationally representative household survey that collects a wide range of data from demographics to maternal and child health. The first round of CDHS was conducted in 1998 and has been repeated since then approximately every fifth year, with the latest survey in 2021–2022. The multi-stage stratifying sampling technique and specifics on the structured questionnaire are presented extensively elsewhere^16^, however the sampling unit for the survey was households. The unit of analysis in our study was under-five children in the CDHS conducted in 2021–2022.

### Outcomes and possible predictor variables

The primary outcome was defined as a child dying before their fifth birthday in the last five years preceding the study. The secondary outcome was defined as a child under the age of five years having fever, acute lower respiratory disease, or diarrhea during the last two weeks preceding the survey. Possible variables that could be associated with the outcome were identified through established frameworks for understanding determinants of child mortality and morbidity^17^, previous studies in Cambodia^7,8,11^ and CDHS data information^16^. A descriptive analysis of the identified variables is presented with weighted counts, accounting for the cluster and sampling design. Of the identified variables, those with less than 30% missing data and where data were captured for all under-five children were used to analyze the primary and secondary outcomes further. This led our multivariable models to include the following variables: twin, birth order of child, previous birth interval of mother, mother age at birth, contraceptive use of mother, mothers’ highest educational level, the number of births in the last five years of the mother, drinking water source, sanitation facility, cooking fuel, electricity, household wealth quintile, household type, geographical region, and health insurance. A detailed description of the variables, including necessary recoding from the CDHS dataset is included in the Supplementary Material (Table S1). Lastly, children with missing data for any of the variables included in the models were excluded from the dataset.

### Statistical analysis

First, the neonatal (from birth to 28 days of life), infant (birth to one year of age) and under-five mortality rates per 1000 live births and their 95% confidence intervals were calculated through Jackknife variance estimator^18^, in line with the established DHS method^19^. Secondly, a survey-weighted univariable and multivariable generalized linear model with a binomial link was used to conduct statistical inference on the primary and secondary outcome probability with robust standard errors clustered at the CDHS cluster level while taking into account the strata^19,20^. Unadjusted odds ratios and 95% confidence intervals were estimated for all variables considered, while adjusted odds ratios and 95% confidence intervals were estimated for the variables included in the respective multivariable models. Large sample two-sided Wald-type statistical tests for the hypothesis that the odds ratios for each predictor were equal to one (no association) were conducted with a type I error fixed at 5%.

A classification random forest machine learning algorithm was applied to identify additional possible predictors and to complement the statistical inference provided by the multivariable logistical regression. In brief, a random forest is a supervised ensemble learning algorithm combining individual decision trees into a random forest^21^. From the original sample, several bootstrap samples are drawn, and an unpruned classification tree is fit for each bootstrap sample. The variable selection for each split in the classification tree is conducted only from a small random subset of predictor variables. In the traditional application of random forest, the split is decided based on the Gini split criterion however, this can lead to decision trees preferring variables with more categories^22,23^. Given the many different categories present in the data, we use a split based on conditional inference framework provided by Hothorn et al.^24^ and built upon by Strobl et al.^25^ that provides unbiased classification decision trees. From the complete forest, the status of the response variable is predicted as an average or majority vote of the predictions of all trees. As such, the algorithm adjusts for the instability of the individual decision trees. In our study, we are not interested in constructing a prediction model, but rather in understanding which of the included variables in the model is most important. Interpreting variable importance from machine learning algorithms can be tricky however, for most datasets and aims, permutation importance provides a robust assessment of variable importance^26^. In short, by randomly permuting the predictor variable *X*_ *j*_, its original association with the response *Y* is broken. When the permuted variable *X*_ *j*_ and the remaining unpermuted predictor variables used to indicate the response, the prediction accuracy (i.e., the number of observations classified correctly) decreases substantially, if the original variable *X*_ *j*_ was associated with the response. Thus, a reasonable measure for variable importance is the difference in prediction accuracy before and after permuting *X*_ *j*_. One important advantage of permutation variable importance is that the measure both covers the non-linear impact of each variable on the prediction accuracy as well as the non-linear multivariable interaction with other predictor variables. In our analysis, the conditional random forest was implemented with default settings and link each observation with the household weight to account for the complex survey design. To assess variable importance, conditional permutation importance was averaged over ten permutations with the threshold level set at a p-value of < 0.05. For details on the statistical properties of conditional decision trees, random forests based on such trees, and permutation importance, we refer the reader to Debeer and Strobl^27^.

The data management and analyses were conducted in R (version 4.1.1)^28^. Child mortality rates were calculated with the chmort function from the DHS.rates package^18^, the complex survey design accounted for with svydesign function and the survey-weighted generalized linear models constructed with a binomial link through svyglm function from the Survey package^20^. The random forest was created through cforest from Party package^29^ and permutation importance calculated with the permimp function from the permimp-package^30^.

### Ethical approval

The survey used in this study has been approved by ICF Institutional Review Board and gained ethical approval from relevant ethical institutional review board in Cambodia. Informed consent was gained from all participants. All analyses were performed in accordance with relevant guidelines and regulations.

## Results

The CDHS included 8153 children under five years, and over the five years before the end of the survey, the reported neonatal mortality rate was 8.40 (95% CI 5.81–10.9) per 1000 live births, infant mortality rate 12.7 (95% CI 9.51–15.8) and under-five mortality rate 19.3 (95% CI 12.3–25.3). In total, 114 (1, 4%) of children died before their fifth birthday, with the majority of deaths (N = 66, 58%) occurring during the neonatal period. During the survey, 1321 (17%) of children had the secondary outcome of fever, acute lower respiratory disease or diarrhea. An overview of the characteristics of the population is provided in Table 1, while univariable analyses of the variables in Table 1 and the primary and secondary outcome is available in Supplementary Material (Table S2). There were no major differences between male and female children, with the exception of a higher proportion of male children being stunted versus female (11% versus 8.4%).

**Table 1:** Characteristics of the study population by sex.

Characteristic	Missing	Overall N = 8019	Male N = 4083	Female N = 3936	p-value
Child/Mother					
Twin* (Yes/No)	0%	1.1% (92/8019)	1.1% (44/4083)	1.2% (48/3936)	0.7
Birth order*	0%				0.8
First		35% (2834/8019)	36% (1460/4083)	35% (1374/3936)	
Second		35% (2813/8019)	35% (1413/4083)	36% (1401/3936)	
Third		19% (1493/8019)	19% (772/4083)	18% (721/3936)	
Fourth and later		11% (878/8019)	11% (438/4083)	11% (440/3936)	
Previous birth interval*	0.2%				0.8
First born		35% (2834/8005)	36% (1460/4079)	35% (1374/3926)	
< 2 years		7.0% (559/8005)	6.8% (278/4079)	7.2% (281/3926)	
2–3 years		21% (1637/8005)	20% (812/4079)	21% (825/3926)	
> 3 years		37% (2975/8005)	37% (1529/4079)	37% (1446/3926)	
Birth weight under 2500g (Yes/No)	41%	6.3% (300/4771)	5.8% (141/2440)	6.8% (159/2331)	0.3
Stunted weight for height (Yes/No)	54%	9.9% (363/3665)	11% (211/1861)	8.4% (152/1804)	0.025
Mother's age at birth*	0%				0.6
< 20 years		8.8% (706/8019)	8.9% (364/4083)	8.7% (342/3936)	
20–34 years		77% (6144/8019)	77% (3144/4083)	76% (3000/3936)	
35–49 years		15% (1169/8019)	14% (575/4083)	15% (595/3936)	
Mother highest educational level*	0%				0.7
No education		11% (884/8019)	12% (471/4083)	10% (413/3936)	
Primary		41% (3294/8019)	41% (1667/4083)	41% (1627/3936)	
Secondary		41% (3258/8019)	40% (1644/4083)	41% (1614/3936)	
Higher		7.3% (583/8019)	7.4% (301/4083)	7.2% (282/3936)	
Contraceptive use* (Yes/No)	0%	64% (5124/8019)	62% (2545/4083)	66% (2579/3936)	0.020
Wanted pregnancy of child	39%				0.5
Yes		81% (3977/4908)	81% (2030/2506)	81% (1947/2402)	
Later		7.9% (387/4908)	8.4% (211/2506)	7.4% (177/2402)	
No		11% (544/4908)	11% (265/2506)	12% (278/2402)	
Births in last five years*	0%				0.5
One		72% (5756/8019)	72% (2932/4083)	72% (2824/3936)	
Two or more		28% (2264/8019)	28% (1151/4083)	28% (1107/3936)	
Household					
Water source*	0%				0.9
Unimproved		9.4% (756/8019)	9.4% (384/4083)	9.5% (373/3936)	
Improved		91% (7263/8019)	91% (3699/4083)	91% (3564/3936)	
Sanitation facility*	0%				0.2
Unimproved		11% (910/8019)	12% (482/4083)	11% (428/3936)	
Improved		89% (7110/8019)	88% (3601/4083)	89% (3508/3936)	
Cooking fuel*	1%				0.5
Electricity/gas		52% (4167/7963)	52% (2102/4056)	53% (2065/3908)	
Kerosine/Coal/Wood or similar		48% (3796/7963)	48% (1953/4056)	47% (1843/3908)	
Electricity* (Yes/No)	0%	91% (7324/8019)	91% (3717/4083)	92% (3607/3936)	0.4
Household wealth quintile*	0%				0.5
Poorest		22% (1732/8019)	23% (919/4083)	21% (814/3936)	
Poorer		19% (1489/8019)	18% (742/4083)	19% (747/3936)	
Middle		19% (1488/8019)	18% (740/4083)	19% (747/3936)	
Richer		21% (1717/8019)	21% (873/4083)	21% (844/3936)	
Richest		20% (1593/8019)	20% (808/4083)	20% (784/3936)	
Household type*	0%				0.5
Urban		39% (3150/8019)	40% (1622/4083)	39% (1528/3936)	
Rural		61% (4869/8019)	60% (2461/4083)	61% (2408/3936)	
Geographical region*	0%				0.2
Phnom Penh		15% (1172/8019)	15% (594/4083)	15% (577/3936)	
Plain		33% (2666/8019)	33% (1338/4083)	34% (1328/3936)	
Great lake		30% (2422/8019)	30% (1227/4083)	30% (1195/3936)	
Coastal		6.0% (479/8019)	6.7% (274/4083)	5.2% (205/3936)	
Mountain/Plateau		16% (1280/8019)	16% (649/4083)	16% (631/3936)	
Health service					
Health insurance* (Yes/No)	0%	25% (1969/8019)	25% (1031/4083)	24% (939/3936)	0.2
Antenatal visits	42%				0.6
0		1.3% (58/4631)	1.4% (34/2366)	1.1% (24/2265)	
1–4		25% (1142/4631)	25% (583/2366)	25% (559/2265)	
> 4		74% (3432/4631)	74% (1750/2366)	74% (1682/2265)	
Place of delivery	39%				0.6
Facility with cesarian section possibility		38% (1842/4908)	38% (959/2506)	37% (884/2402)	
Facility		61% (2973/4908)	60% (1501/2506)	61% (1472/2402)	
Home		1.9% (92/4908)	1.8% (46/2506)	1.9% (47/2402)	
Assisting person during delivery	39%				0.3
Health professional		99% (4846/4908)	99% (2471/2506)	99% (2375/2402)	
Non-health profession		1.3% (62/4908)	1.4% (35/2506)	1.1% (27/2402)	
Any postnatal visit (Yes/No)	42%	38% (1774/4621)	37% (874/2360)	40% (900/2261)	0.12
DTP full vaccination (Yes/No)	40%	75% (3608/4814)	74% (1805/2448)	76% (1802/2366)	0.088
Polio full vaccination (Yes/No)	40%	77% (3725/4832)	76% (1865/2460)	78% (1860/2373)	0.081

All variables are weighted according to household survey weight, while taking into consideration the sampling cluster and strata.

*These variables had data from all under-five children and below 30% missing data and were included in the multivariable model. Region Phnom Penh is the capital city; the Plain region consists of Kampong Cham, Kandal, Prey Veng, Svay Rieng, and Takeo; Great lake region includes Banteay Meanchey, Battambang, Kampong Chhnang, Kampong Thom, Pursat, and Siemreap; Coastal region has Kampot, Kep, Koh Kong, Preah Sihanouk; and Mountain/Plateau region consists of Kampong Speu, Kratie, Preah Vihear, Ratanak Kiri, Mondul Kiri, Stung Treng, Oddar Meanchey, and Pailin. Improved water sources include direct water, piped wells, and covered dug wells. Improved sanitation facilities include toilet or latrine connected with sewage or septic tanks.

**Fig. 1:**
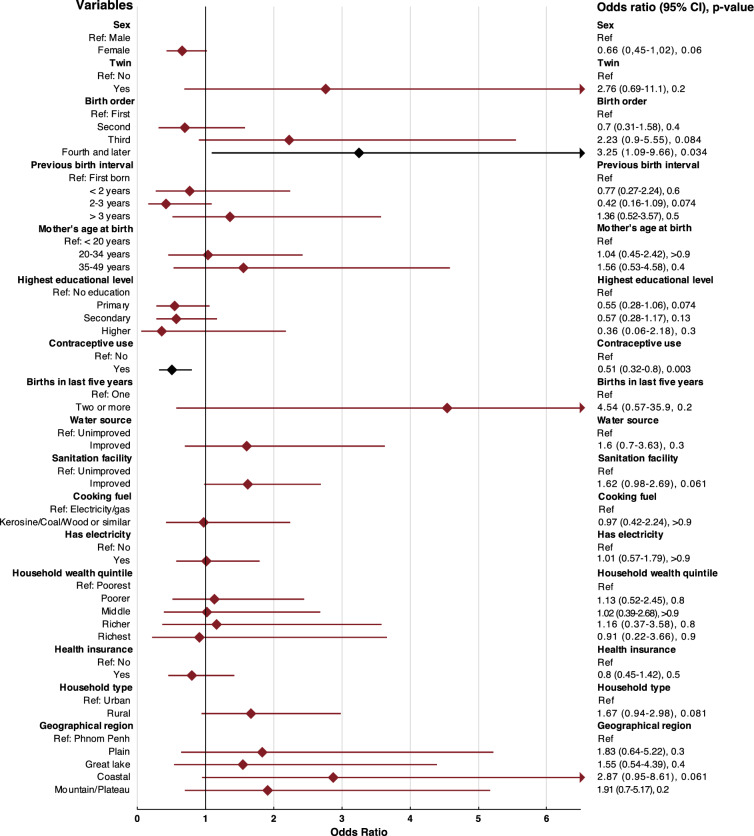
Multivariable logistic regression for the primary outcome of under-five mortality. Black color indicates a statistically significant association.

**Fig. 2:**
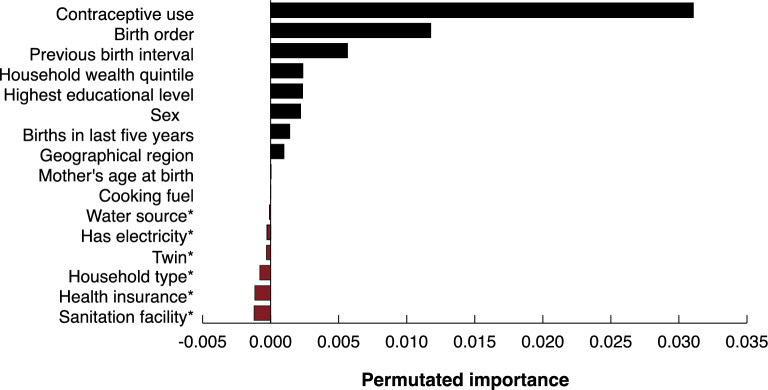
Permutation importance for variables in model for the primary outcome of under-five mortality in Cambodia, ranked from most important to unimportant. *These variables had a value below zero, indicating that the variables were not deemed important for the machine learning algorithm.

For the outcome of under-five mortality, the logistic regression (Fig. 1) indicates that being born fourth or later led to significantly increased odds of mortality (3.25 [95% CI 1.09–9.66], p-value 0.034). A similar tendency was noted for being born third, a twin, living in a rural household and in a geographical region outside of Phnom Penh. On the other hand, if the mother used some form of contraception, there was significantly lower odds of mortality (0.51 [95% CI 0.32–0.80], p-value 0.003). Although not statistically significant, being born a female and mother having any type of education were also indicating a lower odds of mortality. The permuted variable importance of the random forest (Fig. 2) shows contraceptive use to be of the highest importance to the model, followed by birth order, previous birth interval, household wealth quintile, highest educational level of mother, sex of the child, births in the last five years, geographical region, mother´s age at birth and type of cooking fuel while the remainder was deemed not important for the model.

**Fig. 3:**
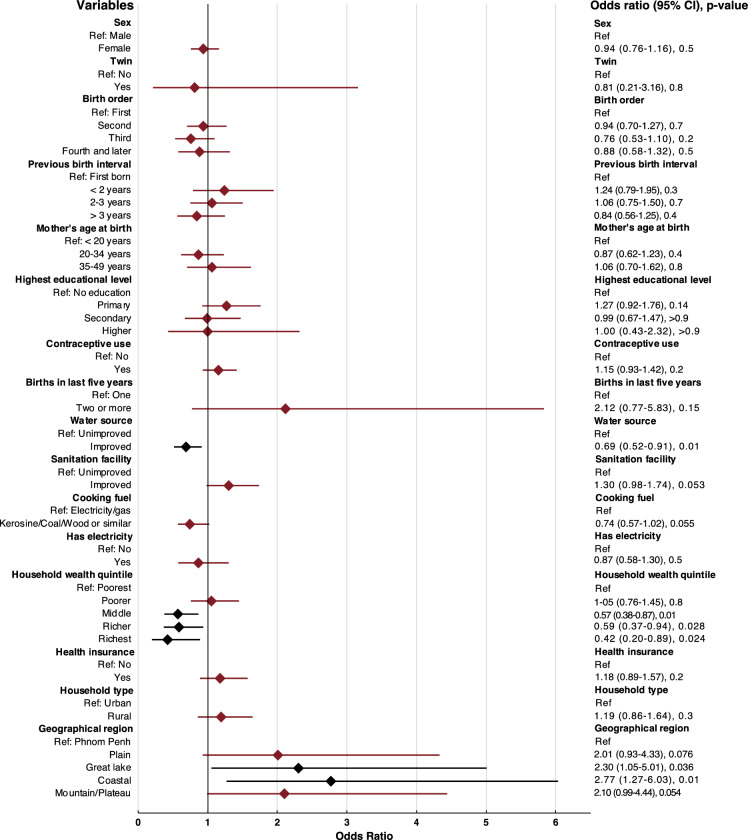
Multivariable logistic regression for the outcome of fever, acute lower respiratory disease or diarrhea any time in the two weeks preceding the survey. Black color indicates a statistically significant association.

**Fig. 4:**
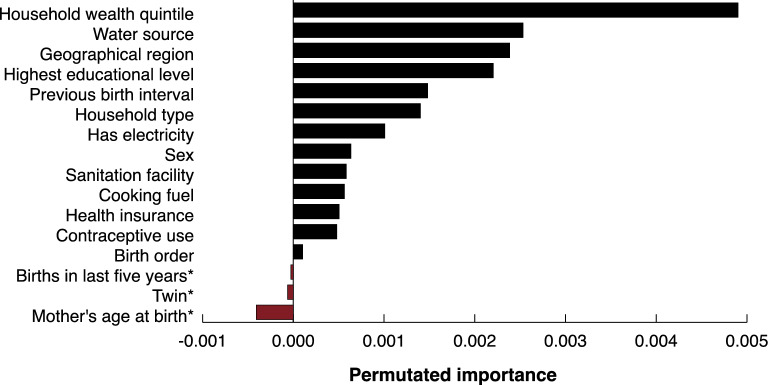
Permutation importance for variables in model for the outcome of fever, acute lower respiratory disease or diarrhea any time in the two weeks preceding the survey, ranked from most important to unimportant. *These variables had a value below zero, indicating that the variables were not deemed important for the machine learning algorithm.

When it comes to the outcome of infectious disease, the multivariable logistic regression results (Fig. 3) indicate that there might be a significantly reduced in odds for children living in households with improved water source (0.69 [95% CI 0.52–0.91], p-value 0.01), being in the middle (0.57 [95% CI 0.38–0.87], p-value 0.01), richer (0.59 [95% CI 0.37–0.94], p-value 0.028) or richest (0.42 [95% CI 0.20–0.89], p-value 0.024) wealth quintiles. None of the child-specific variables had a statistically significant association with the infectious disease outcome. There were a significantly increased odds if the child lived in the Coastal (2.30 [95% CI 1.05–5.01], p-value 0.036) or Great Lake (2.77 [95% CI 1.27–6.03], p-value 0.01) geographical regions. For the random forest (Fig. 4), the most important variables were deemed to be household wealth quintile, water source, geographical region and highest educational level of the mother with the remaining being important for the model except for the number of births in the last five years, if the child was a twin or not and the mothers age at birth.

## Discussion

In this study examining the possible determinants of child mortality and child morbidity from infectious diseases in Cambodia in 2021–2022 we show a continued decline of the under-five mortality rate with the majority of under-five deaths occurring during the neonatal period, and that infectious diseases contribute to significant morbidity burden. Including both traditional multivariable logistical regression and machine learning analysis, variables that were significantly associated with the outcomes also had a relatively high permutated variable importance in the random forest such as contraceptive use and household wealth quintile. Indeed, household wealth quintile, highest educational level of the mother and previous birth order seemed in the random forest model to be important for both primary and secondary outcomes, indicating that there are similar determinants of under-five child mortality and child morbidity from infectious diseases in Cambodia.

In our study, we found that contraceptive use was significantly associated with a reduced odds of under-five mortality, and in the study population roughly two thirds of the mothers used any type of contraception. This is in line with the analysis done by Um and Heng based on the 2014 CDHS^8^ which also found contraceptive use to be associated with lower under-five mortality albeit with tendency for a bit lower odds ratio than what we found (0.51 [95% CI 0.32–0.80] versus (0.30 [95% CI 0.18–0.52]). Even though the multivariable models included variables that to some extent account for the mother´s agency and empowerment, such as the education level, it is likely that the association between contraceptive use of the mother and odds of under-five mortality suffer from confounding. It has previously been shown that contraceptive use is closely linked to how empowered a woman is within the household^31^ and that higher attained education level of the woman^32^ improves the likelihood of contraceptive use in Cambodia. Contraceptive use allows mothers to space and plan pregnancies, leading to lower risk of unwanted pregnancies and has been shown to reduce infant mortality rates^33^. In Cambodia, women often do not have the full autonomy of choice when it comes to contraceptive method, and face cultural and practical barriers to accessing modern reversible contraceptive methods^34^. Given the lack of data on health service seeking pattern in our model, contraceptive use might also be indicative of health literacy and health seeking behavior^32^ among mothers which could serve as a protective factor against under-five mortality. Being born fourth or later led in our analysis to a significantly increased odds of under-five mortality however it should be noted that the confidence interval is quite broad. Similarly, the birth interval was deemed third most important by the random forest. Both birth order and birth interval had not previously been identified as associated with under-five mortality in Cambodia^8,11^ and might reflect the changing demography of Cambodia over time with the relatively fewer poorer households having more children. Beyond Cambodia, having a high birth order have been found to be associated with increased risk of under-five mortality in low and middle-income settings^35,36^, while also increase the risk of undernutrition^37,38^ and even in high-income settings the effects of birth order continues throughout the life course^39^.

Similar to global burden of disease estimates^9^, our results indicate a significant burden of infectious disease among children under-five years in Cambodia. When exploring determinants of infectious disease among children under five, we found that households who had an improved water source had a lower odds of infectious disease. This is in line with the findings from CDHS 2014 by Vong et al.^11^ that showed a higher risk of diarrhea among children living in households with unimproved water source. Water quality and risk of infectious disease among children is well-established in low- and middle-income countries^40^. With climate change leading to increased precipitation in many settings the importance of household access to quality water and sanitation to protect children from infectious disease has become clear^41^. Unsurprisingly, we found that children living in households belonging to the higher wealth quintiles (middle, rich or richest) had substantially lower odds of infectious disease. With improved but unequal living standards and economic growth over the last two decades, Cambodia has experienced a shift in under-five mortality and morbidity, with neonatal mortality driving under-five mortality and infectious disease among children primarily affecting poor and vulnerable households^42–45^. Children living in the coastal (Kampot, Kep, Koh Kong and Preah Sihanouk) or great lake (Banteay Meanchey, Battambang, Kampong Chhnang, Kampong Thom, Pursat and Siemreap) regions had a higher odds of infectious disease. The prevalence of infectious disease pathogens among children in different regions of Cambodia is not known, however children living in proximity to water bodies in these regions might be more exposed to spread of infectious disease pathogens^46^. Additionally, these regions are also prone to cyclones and flooding^47^ which might further lead to increased transmission of infectious diseases. Overall, the result from our study depicts how already vulnerable children in certain geographies are more at risk from infectious disease in Cambodia.

We find that under-five mortality and morbidity due to infectious diseases are associated with characteristics of the mother and household. Specifically, empowering women and promoting safe contraceptive use and family planning programs might further reduce under-five mortality in Cambodia. Since 2019, the Ministry of Health in Cambodia has implemented a cash transfer scheme for pregnant women from families with an IDPoor card^5^ to further improve maternal and child health outcomes in Cambodia, including reducing mortality. Women are eligible to receive three stages of support including: 10 USD every antenatal care visit up to four visits, an additional one-time payment of 50 USD for new mothers after delivery in a health facility and 10 USD for each post-delivery check-ups for themselves and their children up to ten times until their children are two-years old^48^. Additionally, investing in quality water and sanitation systems for all along with recognizing the health disparities between households should be key when designing public health programs targeting infectious diseases. There is a lack of data on a district level on the prevalence of different infectious diseases affecting children, development of local surveillance systems and making the data publicly available should be prioritized. Our study showcases how child mortality and morbidity from infectious disease are linked to many sectors beyond the health sector, and that a random forest analysis can complement traditional statistical approaches to illuminate factors that might be influencing outcomes not fully captured in traditional statistical methods^49^. Acting on synergies and handling tradeoffs between sectors is key to put child health in the center of sustainable development^50^. Multisectoral programs that tackle multiple vulnerabilities, such as IDPoor^5^, holds promise to further accelerate progress which will be necessary if Cambodia is to reach the Sustainable Development Goal target 3.2 of ending preventable deaths of newborns and children under five years of age.

The DHS surveys follows a stringent data collection process and has provided high quality data on sociodemographic factors for more than three decades^51^. However, analysis based on DHS has its limitations. First, even though the CDHS follows a highly standardized approach, the respondents in the survey might have recall or omission bias which could skew the results. Secondly, for information regarding the prenatal, delivery and postnatal period only data for children below three years of age is included in the questionnaire resulting in all children three years or above are missing data on these variables. Additionally, among these variables there were a significant amount of missing data for children who died, making it methodologically questionable to construct multivariable models for this sub-group alone. Moreover, the lack of available variables representing the mother´s empowerment or health service utilization limits our understanding of the associations found. Thirdly, it is not possible to assess the underlying infectious pathogen causing diarrhea, cough or fever which limits the possibility to decipher pathogen-specific determinants of under-five infectious disease morbidity. Fourthly, when it comes to the statistical analysis, random forest cannot fully incorporate the complex survey design structure of the data even though survey weights can be included to mitigate this problem. An important consideration is that given the different assumptions of traditional logistical regression and machine learning algorithms such as random forest, comparing the findings between the logistical regression and the random forest should be done with caution particularly since random forest cannot make estimates of inference or the direction of the association. Although we present a variable importance measure, the direction of the relationship is not incorporated, and it is not possible to untangle why the random forest deem certain variables unimportant or important. In our study, we apply both methods in a complimentary manner in order to explore possible associations rather than to compare the approaches or assert causality. For instance, the random forest might provide a more nuanced view on associations and provide a starting point of further detailed exploration of variables that were seen as important by the random forest but not independently associated with the outcome in the logistical regression. Altogether, the strengths and limitations of this study reflects the complexities of trying to assert real-world associations in a data and analysis-limited environment.

## Conclusion

The majority of under-five deaths in Cambodia occurred during the neonatal period, and under-five mortality was significantly associated with contraceptive use of the mother and the birth order of the child. Child morbidity due to infectious disease was associated with water source, household wealth quintile and geographical region. The findings showcase how closely related child mortality and morbidity due to infectious disease are to broader social development in Cambodia and the importance of accelerating progress to end preventable child mortality and morbidity.

### Supplementary Information

Supplementary Tables.

## Data Availability

The datasets generated and/or analyzed are available in the Demographic and Health Service (DHS) Programme repository, at: https://dhsprogram.com/data/using-datasets-for-analysis.cfm.
