# Supplementary material for: Exploring the determinants of under-five mortality and morbidity from infectious diseases in Cambodia—a traditional and machine learning approach
Source: Sci Rep. 2024 Aug 27;14:19847. doi: 10.1038/s41598-024-70839-z (PMC11350148; doi:10.1038/s41598-024-70839-z)
Supplement: Supplementary file 1 — Supplementary Tables. [file 41598_2024_70839_MOESM1_ESM.pdf]

Supplementary Material

Exploring the determinants of under-five mortality and morbidity from infectious diseases in Cambodia – a traditional and machine learning approach

Authors: Daniel Helldén[1,2], Serey Sok[3], Alma Nordenstam[1], Nicola Orsini[1], Helena Nordenstedt[1,4], Tobias Alfvén[1,5]

Affiliations:
[1]Department of Global Public Health, Karolinska Institutet, Stockholm, Sweden

[2] Astrid Lindgren Children's Hospital, Karolinska University Hospital, Stockholm, Sweden

[3] Research Office, Royal University of Phnom Penh, Phnom Penh, Cambodia

[4] Department of Medicine and Infectious diseases, Danderyd University Hospital, Stockholm, Sweden

[5]Sachs' Children and Youth Hospital, Stockholm, Sweden

Table of Contents

Table S1. Variables and recoding from the Cambodian Demographic Health Survey 2021-2022.

Table S2. Univariable estimated odds ratios and 95% confidence intervals for all variables in Table 1 for under-five mortality and infectious diseases using a survey-weighted multivariable logistic regression.

Table S1. Variables and recoding from the Cambodian Demographic Health Survey 2021-2022.

Variable	Available for alive and dead children	Data collected for children <3 years or <5 years	Missing	Definition in DHS	Recoding for analyses
**Outcomes**					
**Primary Outcome: Under-five mortality**	Available	<5 years	0%	Numeric.<br>Child under five year is alive (0=No, 1=Yes)	Binary.<br>Under five death (1=Yes, 0=Yes)
**Secondary outcome:**<br>**Infectious disease morbidity**	Available	<5 years	0%	Numeric.<br>1) Child having fever any time in the 2 weeks before the survey (1=Yes, 0=No)<br>2) Child having diarrhea any time in the 2 weeks before the survey (1=Yes, No=0)<br>3) Child having acute respiratory symptoms any time in the 2 weeks before the survey (1=Yes, No=0)	Binary:<br>Symptoms of infectious disease (fever, diarrhea or acute respiratory symptoms) any time in the 2 weeks before the survey (1=Yes, 0=No)
**Child/Mother**					
**Sex***	Available	<5 years	0%	Numeric.<br>Sex of child (1=Male, 2=Female)	Factor.<br>Sex of child (0=Male, 1=Female)
**Twin***	Available	<5 years	0%	Numeric.<br>Born a twin (0=No, 1=Yes)	Factor.<br>Born a twin (0=No, 1=Yes)
**Birth order***	Available	<5 years	0%	Numeric.<br>Birth order of the child.	Factor.<br>Birth order of the child. (1=First, 2= Second, 3=Third, 4=Fourth and later)
**Previous birth interval***	Available	<5 years	0.2%	Numeric.<br>Length of interval in months between the birth of	Factor.<br>Previous birth interval. (0=First born, 1=<2 years, 2=2-3 years, 3 = >3 years).

				the previous child and the current (if child was not first born).	
**Birth weight under 2500g**	Available	<3 years	41%	Numeric.<br>Birth weight of child, either from written card or from mother´s recall.	Factor.<br>Birth weight under 2500g (0=No, 1=Yes)
**Stunted weight for height**	Not available	<5 years	54%	Numeric.<br>Weight for height standard deviations.	Factor.<br>Stunted defined as -2 or bellow standard deviation. (0=No, 1=Yes)
**Mother's age at birth***	Available	<5 years	0%	Numeric.<br>Age of the mother at the birth of the child in years.	Factor.<br>Age of the mother at the birth of the child in years (1= <20 years, 2= 20-34 years, 3=35-49 years)
**Mother highest educational level***	Available	<5 years	0%	Factor.<br>Highest educational level of mother (No education, Primary, secondary, Higher)	No recoding necessary.
**Contraceptive use***	Available	<5 years	0%	Factor.<br>Current contraceptive use (0=Not using, 1=pill, 2=iud, 3=injections, 4= diaphragm, 5= male condom, 6= female sterilization,7=male sterilization, 8=periodic abstinence, 9= withdrawal, 10=other traditional, 11= implants/Norplant, 12= prolonged abstinence, 13= lactational amenorrhea (lam), 14=female condom, 15=foam or jelly, 16=emergency contraception, 17=other modern method, 18 standard days method (sdm), 19= specific method 1, 20=specific method 2	Factor.<br>Current contraceptive use (0=Not using, 1= Any contraceptive use).

**Wanted pregnancy of child**	Available	<3 years	39%	Factor.<br>If the child was wanted or not (1=Yes, 2=Later, 3=No)	No recoding necessary.
**Births in last five years***	Available	<5 years	0%	Numeric.<br>Number of births of the mother in the last five years.	Factor.<br>Number of births of the mother in the last five years (1=One, 2= Two or more)
**Household**					
**Water source***	Available	<5 years	0%	Factor.<br>Type of water source (10=piped water, 11=piped into dwelling, 12=piped to yard/plot, 13=piped to neighbor, 14=public tap/standpipe, 20=tube well water, 21=tube well or borehole, 31=protected well, 32=unprotected well, 41=protected spring, 42=unprotected spring, 43=river/dam/lake/ponds/ stream/canal/irrigation channel, 51=rainwater, 61=tanker truck, 62= cart with small tank, 71=bottled water	Factor.<br>Type of water source.<br>0=Unimproved (32=unprotected well, 42=unprotected spring, 43=river/dam/lake/ponds/ stream/canal/irrigation channel)<br>1=Improved (10=piped water, 11=piped into dwelling, 12=piped to yard/plot, 13=piped to neighbor, 14=public tap/standpipe, 20=tube well water, 21=tube well or borehole, 31=protected well, 41=protected spring, 51=rainwater, 61=tanker truck, 62= cart with small tank, 71=bottled water)
**Sanitation facility***	Available	<5 years	0%	Factor.<br>Type of sanitation facility (10=flush toilet, 21= ventilated improved pit latrine, 22=pit latrine with slab, 23=pit latrine without slab/open pit, 31=No facility/bush/field, 41=composting toilet, 42=bucket toilet, 43=hanging toilet/latrine)	Factor.<br>Type of sanitation facility.<br>0=Unimproved (23=pit latrine without slab/open pit, 31=No facility/bush/field, 42=bucket toilet, 43=hanging toilet/latrine)<br>1=Improved (10=flush toilet, 21= ventilated improved pit latrine, 22=pit latrine with

					slab, 41=composting toilet)
**Cooking fuel***	Available	<5 years	1%	Factor.<br>Type of cooking fuel in household (1=electricity, 2=solar energy, 3=liquefied petroleum gas (lpg)/cooking gas, 4= piped natural gas, 5= biogas, 6= alcohol/ethanol, 7= gasoline/diesel, 8= kerosene/paraffin, 9= coal/lignite, 10=charcoal, 11= wood, 12=straw/shrubs/grass, 13=agricultural crop, 14= animal dung/waste, 15=processed biomass (pellets) or woodchips, 16=garbage/plastic, 17=sawdust.	Factor.<br>Type of cooking fuel in household.<br>0=Electricity/gas(1=electricity, 2=solar energy, 3=liquefied petroleum gas (lpg)/cooking gas, 4= piped natural gas, 5= biogas)<br>1= Kerosine/Coal/Wood or similar (6= alcohol/ethanol, 7= gasoline/diesel, 8= kerosene/paraffin, 9= coal/lignite, 10=charcoal, 11= wood, 12=straw/shrubs/grass, 13=agricultural crop, 14= animal dung/waste, 15=processed biomass (pellets) or woodchips, 16=garbage/plastic, 17=sawdust.
**Electricity***	Available	<5 years	0%	Factor.<br>Household has electricity (0=No, 1=Yes)	No recoding necessary.
**Household wealth quintile***	Available	<5 years	0%	Factor.<br>Household wealth quintile (Poorest, Poorer, middle, Richer, Richest).	No recoding necessary.
**Household type***	Available	<5 years	0%	Factor.<br>Houshold type (Urban, Rural).	No recoding necessary.
**Geographical region***	Available	<5 years	0%	Factor.<br>Geographical province of household (Phnom Penh, Kampong Cham, Kandal, Prey Veng, Svay Rieng, Takeo, Banteay Meanchey, Battambang, Kampong Chhnang, Kampong Thom, Pursat, Siemreap, Kampot, Kep, Koh Kong, Preah Sihanouk, Kampong Speu, Kratie, Preah Vihear,	Factor.<br>Geographical region of household (1=Phnom Penh, 2=Plain region=Kampong Cham, Kandal, Prey Veng, Svay Rieng, Takeo, 3=Great lake region =Banteay Meanchey, Battambang, Kampong Chhnang, Kampong Thom, Pursat, Siemreap, 4=Coastal region=Kampot, Kep,

				Ratanak Kiri, Mondul Kiri, Stung Treng, Oddar Meanchey, Pailin)	Koh Kong, Preah Sihanouk, 5= Mountain/Plateau region=Kampong Speu, Kratie, Preah Vihear, Ratanak Kiri, Mondul Kiri, Stung Treng, Oddar Meanchey, Pailin.
**Health service**					
**Health insurance*** (Yes/No)	Available	<5 years	0%	25% (1969 / 8019)	25% (1031 / 4083)
**Antenatal visits**	Available	<3 years	42%	Numeric.<br>Number of antenatal visits before birth of the child.	Factor.<br>Number of antenatal visits before birth of the child (0=0, 1=1-4, 2= >4)
**Place of delivery**	Available	<3 years	39%	Factor.<br>Place of delivery of the child (10=home, 21=national hospital, 22=provincial hospital, 23=reference hospital, 24=health center, 25=health post, 26=family clinic, 27=public mobile clinic, 28=other public, 31=private hospital, 32=private clinic, 33=consultation and treatment, 34=general care room, 36=private mobile clinic, 37=other private medical, 41=ngo hospital, 42=ngo sector, 46=ngo other)	Factor.<br>Place of delivery of the child 1=Facility with cesarian section possibility=21=national hospital, 22=provincial hospital, 23=reference hospital, 31=private hospital, 41=ngo hospital<br>2=Health facility24=health center, 25=health post, 26=family clinic, 27=public mobile clinic, 28=other public, 32=private clinic, 33=consultation and treatment, 34=general care room, 36=private mobile clinic, 37=other private medical, 42=ngo sector, 46=ngo other<br>3=10=home
**Assisting person during delivery**	Available	<3 years	39%	Factor.<br>Doctor assisting delivery (0=No, 1=Yes).<br>Nurse/midwife assisting delivery (0=No, 1=Yes).<br>Auxillary midwife assisting delivery (0=no, 1=Yes)	Factor.<br>Assisting person during delivery (0=non-health professional, 1=doctor, nurse/midwife or auxillary midwife).

**Any postnatal visit**	Available	<3 years	42%	Numeric.<br>Number of postnatal visits.	Factor.<br>Any postnatal visit (0=No, 1=Yes)
**DTP full vaccination** (Yes/No)	Missing	<5 years	40%	Factor.<br>Received 3rd DTP dose (0=No, 1=vaccination on card, 2=reported by mother)	Factor.<br>Received 3rd DTP dose (0=No, 1=Yes)
**Polio full vaccination** (Yes/No)	Missing	<5 years	40%	Factor.<br>Received 3rd Polio dose (0=No, 1=vaccination on card, 2=reported by mother)	Factor.<br>Received 3rd Polio dose (0=No, 1=Yes)

Footnotes: * These variables are included in the multivariable model. Note that the DHS was compiled in accordance with the DHS standards (https://dhsprogram.com/publications/publication-DHSG4-DHS-Questionnaires-and-Manuals.cfm)

Table S2. Univariable estimated odds ratios and 95% confidence intervals for all variables in Table 1 for under-five mortality and infectious diseases using a survey-weighted logistic regression.

	**Under-five mortality**		**Infectious disease (fever, acute respiratory infection or diarrhea)**	
**Variable**	**Odds ratio (95% Confidence interval)**	**p-value**	**Odds ratio (95% Confidence interval)**	**p-value**
**Child/Mother**				
**Sex***				
Ref: Male				
Female	0.65 (0.43-0.99)	0.044	0.92 (0.79-1.06)	0.2
**Twin***				
Ref: No				
Yes	5.38 (1.40-20.7)	0.014	1.84 (0.99-3.41)	0.054
**Birth order***				
Ref: First				
Second	0.74 (0.43-1.29)	0.3	1.01 (0.86-1.19)	>0.9
Third	1.55 (0.88-2.74)	0.13	1.06 (0.86-1.31)	0.6
Fourth and later	2.58 (1.34-4.97)	0.005	1.08 (0.83-1.40)	0.6
**Previous birth interval***				
Ref: First born				
<2 years	2.29 (1.17-4.49)	0.016	0.96 (0.69-1.33)	0.8
2-3 years	0.69 (0.32-1.50)	0.4	0.93 (0.72-1.19)	0.6
>3 years	1.14 (0.64-2.04)	0.7	1.07 (0.90-1.27)	0.4
**Birth weight under 2500g**				
Ref: No				
Yes	14.9 (7.51-29.7)	<0.001	1.19 (0.85-1.66)	0.3
**Stunted weight for height**[1]				
Ref: No				
Yes			1.09 (0.76-1.55)	0.6
**Mother's age at birth***				
Ref: < 20 years				

20-34 years	1.13 (0.52-2.41)	0.8	0.82 (0.65-1.04)	0.10
35-49 years	2.46 (1.01-5.99)	0.048	0.83 (0.62-1.09)	0.2
**Highest educational level***				
Ref: No education				
Primary	0.45 (0.24-0.84)	0.012	1.04 (0.83-1.30)	0.7
Secondary	0.38 (0.20-0.73)	0.004	0.80 (0.62-1.02)	0.073
Higher	0.14 (0.03-0.59)	0.007	0.44 (0.27-0.70)	<0.001
**Contraceptive use***				
Ref: No				
Yes	0.44 (0.27-9.70)	<0.001	1.25 (1.08-1.45)	0.003
**Wanted pregnancy of child**				
Ref: Then				
Later	1.29 (0.46-3.66)	0.6	1.63 (1.20-2.21)	0.002
No more	2.03 (0.76-5.44)	0.2	1.35 (0.99-1.83)	0.055
**Births in last five years***				
Ref: One				
Two or more	9.92 (4.29-23.0)	<0.001	1.29 (0.73-2.27)	0.4
**Household**				
**Water source***				
Ref: Unimproved				
Improved	1.41 (0.69-2.87)	0.3	0.68 (0.54-0.86)	0.001
**Sanitation facility***				
Ref: Unimproved				
Improved	0.86 (0.50-1.47)	0.6	0.91 (0.73-1.13)	0.4
**Cooking fuel***				
Ref: Electricity/gas				
Kerosine/Coal/ Wood or similar	0.76 (0.49-1.19)	0.2	0.95 (0.65-1.31)	0.3
**Electricity***				
Ref: No				

Yes	0.65 (0.39-1.09)	0.10	0.73 (0.56-0.96)	0.025
**Household wealth quintile***				
Ref: Poorest				
Poorer	0.77 (0.40-1.47)	0.4	1.24 (1.01-1.52)	0.044
Middle	0.80 (0.46-1.40)	0.4	0.82 (0.66-1.03)	0.084
Richer	0.78 (0.41-1.49)	0.4	0.76 (0.59-0.96)	0.024
Richest	0.36 (0.13-1.04)	0.059	0.38 (0.26-0.54)	<0.001
**Household type***				
Ref: Urban				
Rural	2.01 (1.15-3.53)	0.014	1.70 (1.38-2.10)	<0.001
**Geographical region***				
Ref: Phnom Penh				
Plain	2.16 (0.54-8.62)	0.3	2.63 (1.69-4.08)	<0.001
Great lake	2.20 (0.54-8.96)	0.3	2.57 (1.64-4.02)	<0.001
Coastal	3.30 (0.73-14.9)	0.12	1.87 (1.13-3.10)	0.014
Mountain/Plateau	2.46 (0.62-9.73)	0.2	2.25 (1.44-3.51)	<0.001
**Health service**				
**Health insurance***				
Ref: No				
Yes	0.68 (0.37-1.24)	0.2	0.91 (0.74-1.12)	0.4
**Antenatal visits**				
Ref: 0				
1-4	0.98 (0.12-7.76)	>0.9	0.85 (0.39-1.84)	0.7
>4	0.39 (0.05-3.08)	0.4	0.59 (0.28-1.24)	0.2
**Place of delivery**				
Ref: Facility with cesarian section possibility				
Facility	1.40 (0.68-2.87)	0.4	1.28 (1.06-1.56)	0.012
Home	3.97 (1.08-14.6)	0.038	2.35 (1.44-3.83)	<0.001
**Assisting person during delivery**				

Ref: Health professional				
Non-health profession	5.43 (1.71-17.3)	0.004	2.06 (1.13-3.73)	0.018
**Any postnatal visit**				
Ref: No				
Yes	0.71 (0.30-1.66)	0.4	0.93 (0.77-1.12)	0.5
**DTP full vaccination**[1]				
Ref: No				
Yes			1.24 (1.01-1.53)	0.044
**Polio full vaccination**[1]				
Ref: No				
Yes			1.22 (0.99-1.50)	0.066

Footnotes: * These variables are included in the multivariable model. [1] These variables did not have any data for children who died. Region Phnom Penh is the capital city; the Plain region consists of Kampong Cham, Kandal, Prey Veng, Svay Rieng, and Takeo; Great lake region includes Banteay Meanchey, Battambang, Kampong Chhnang, Kampong Thom, Pursat, and Siemreap; Coastal region has Kampot, Kep, Koh Kong, Preah Sihanouk; and Mountain/Plateau region consists of Kampong Speu, Kratie, Preah Vihear, Ratanak Kiri, Mondul Kiri, Stung Treng, Oddar Meanchey, and Pailin. Improved water sources include direct water, piped wells, and covered dug wells. Improved sanitation facilities include toilet or latrine connected with sewage or septic tanks.
